# Supplementary material for: Disruption of NIPBL/Scc2 in Cornelia de Lange Syndrome provokes cohesin genome-wide redistribution with an impact in the transcriptome
Source: Nat Commun. 2021 Jul 27;12:4551. doi: 10.1038/s41467-021-24808-z (PMC8316422; doi:10.1038/s41467-021-24808-z)
Supplement: Supplementary file 1 — Supplementary Files [file 41467_2021_24808_MOESM1_ESM.pdf]

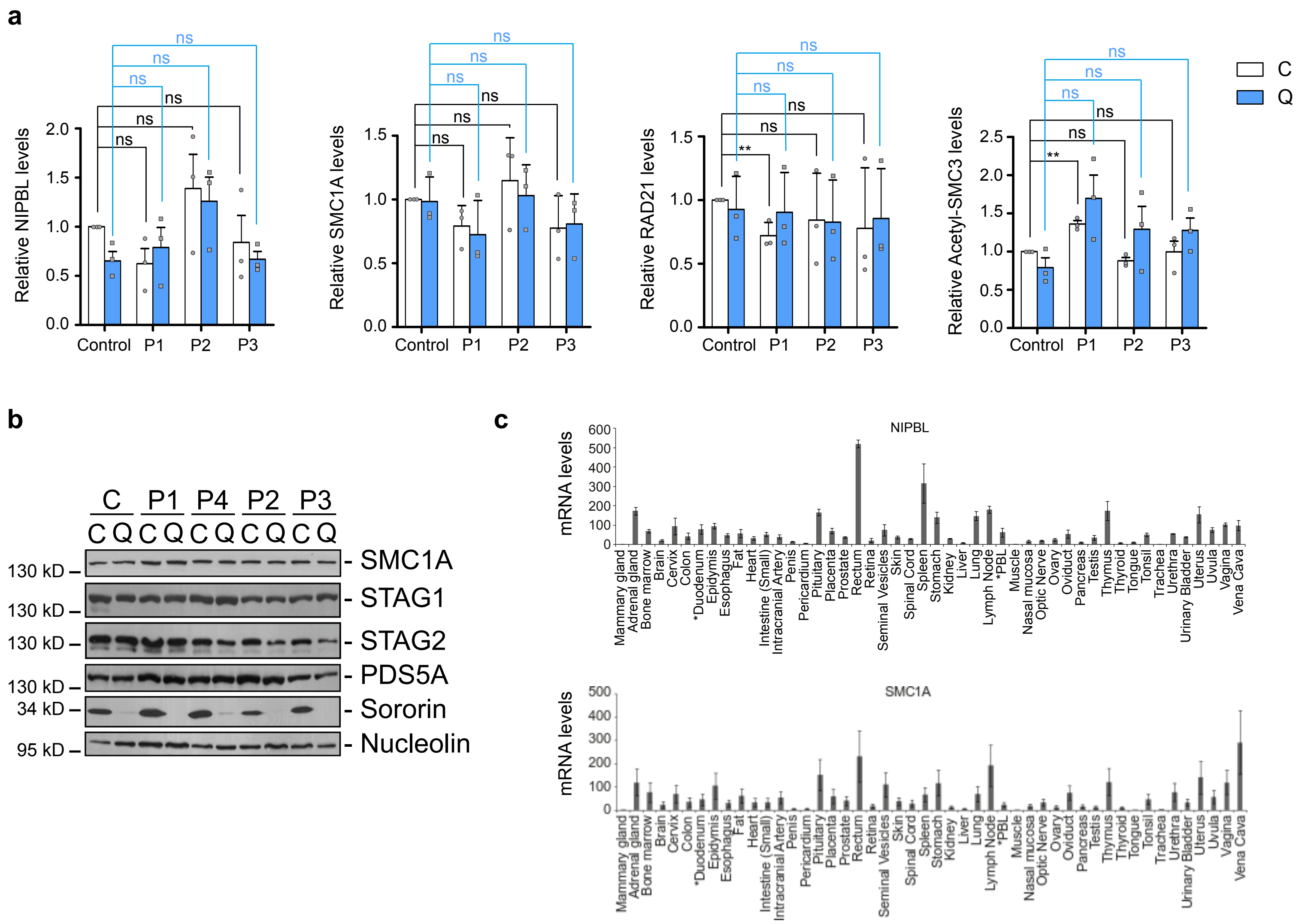

Supplementary Figure 1. Cohesin and NIPBL protein levels are not affected in CdLS-derived cells. (a) Quantification of protein levels of NIPBL, SMC1A, RAD21 and acetyl-SMC3 showed in Figure 1a in control and three CdLS patient (P1-3) derived cells under cycling (C, white) and quiescent (Q, blue) conditions in three biologically independent experiments. The graphs show the amount of each protein relative to actin levels. Means and SEMs are shown. Two-sided unpaired student's *t*-test (***p* <0.01; **p* <0.05). **(b)** Comparison of protein levels of SMC1A, STAG1, STAG2, PDS5A and sororin in control and four CdLS patient (P1-4) derived cells under cycling (C) and quiescent (Q) conditions. Nucleolin was used as loading control. Representative blots from one biological triplicate are shown. **(c)** mRNA expression levels of *NIPBL* and *SMC1A* measured by qPCR in a TissueScan, Human normal cDNA array (Human major Tissue qPCR array containing first-strand DNA from 48 tissues, HMRT304, Origene) in three biologically independent experiments. Means and SEMs are shown.

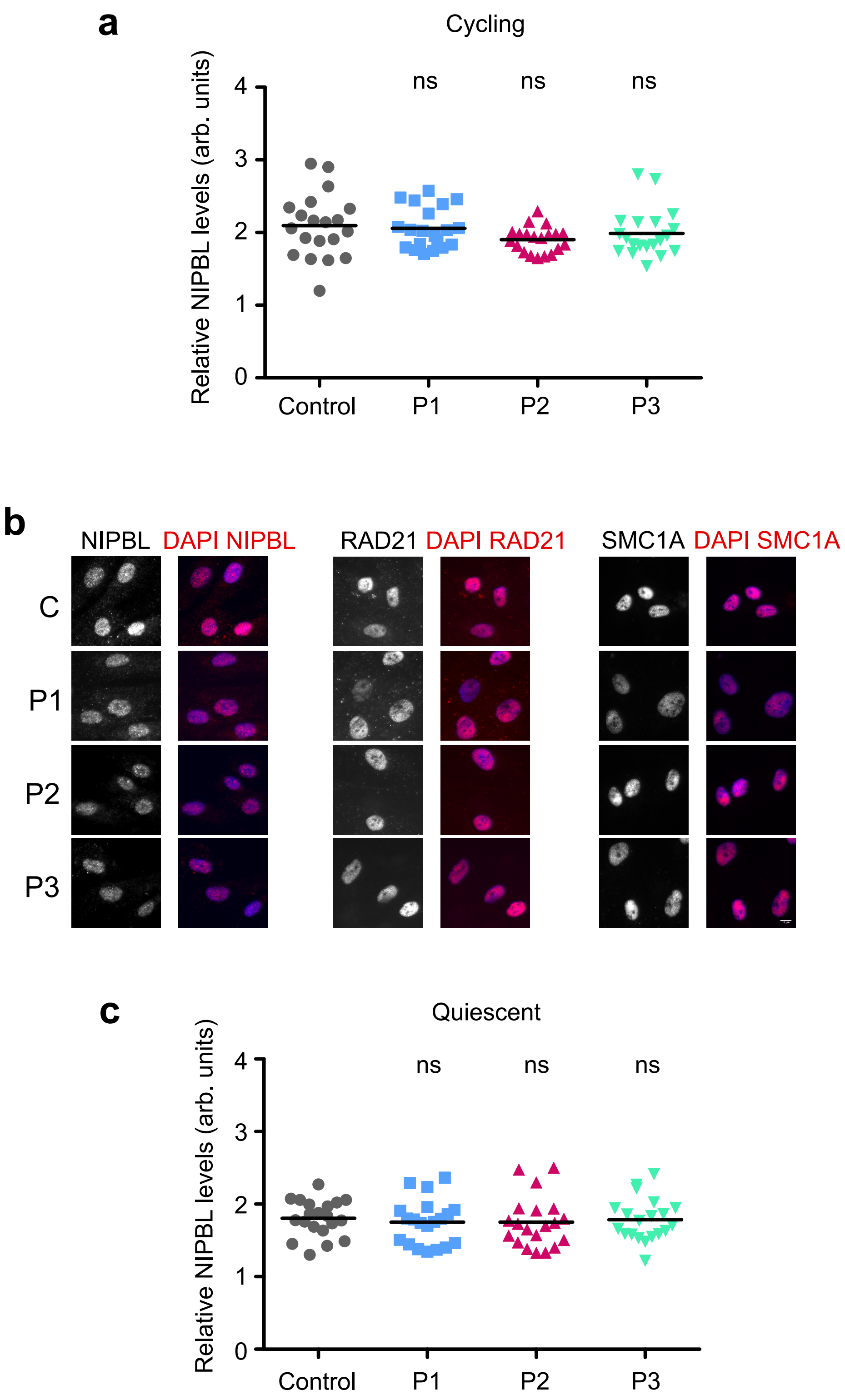

Supplementary Figure 2. The localization of NIPBL and cohesin is not altered in quiescent CdLS-derived cells. **(a)** Quantification of fluorescence levels of NIPBL showed in Figure 1c in control (grey) and three CdLS patient (P1, blue; P2, purple; P3, green)-derived cells (n=20 cells/sample over more than 3 biologically independent experiments) under cycling conditions. Means and SEMs are shown. Two-sided unpaired student's *t*-test. **(b)** Nuclear localization of NIPBL, RAD21 and SMC1A were monitored by immunofluorescence of a control (C) and three CdLS patient (P1-3)-derived fibroblasts under quiescent conditions. Nuclei were stained using DAPI. Representative images from one biologically independent replicate are depicted. Scale bar, 10 μm. **(c)** Quantification of fluorescence levels (from (b)) of NIPBL in control (grey) and three CdLS patient (P1, blue; P2, purple; P3, green)-derived cells (n=20 cells/sample over more than 3 biologically independent experiments) under quiescent conditions. Means and SEMs are shown. Two-sided unpaired student's *t*-test.

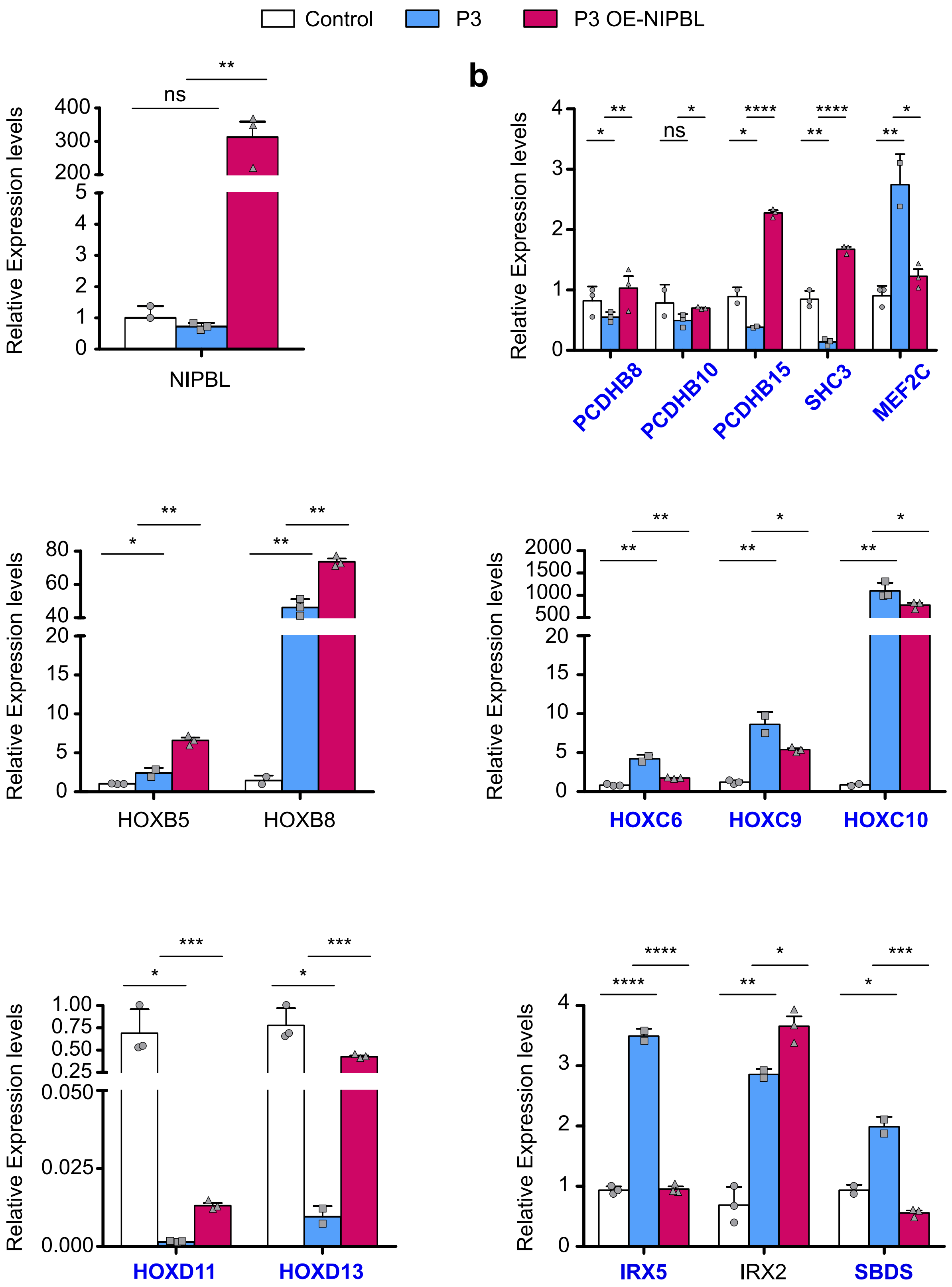

Supplementary Figure 3. Overexpression of NIPBL restored the gene expression in *NIPBL*-deficient derived cells. The mRNA expression levels of *NIPBL* **(a)**, nervous system development genes **(b)**, and embryonic development genes **(c)** were analyzed in a control (white) and CdLS patient-derived fibroblasts in basal conditions (P3, blue) or overexpressing *NIPBL* (P3 *OE-NIPBL*, purple) by reverse transcription-qPCR. The graphs show the amount of transcript of each gene relative to that in the control. Genes which expression is recovered are marked in dark blue. Means and SEMs were calculated from biological triplicates. Two-sided unpaired student's t-test (**** p <0.0001; *** p <0.001; ** p <0.01; * p <0.05).

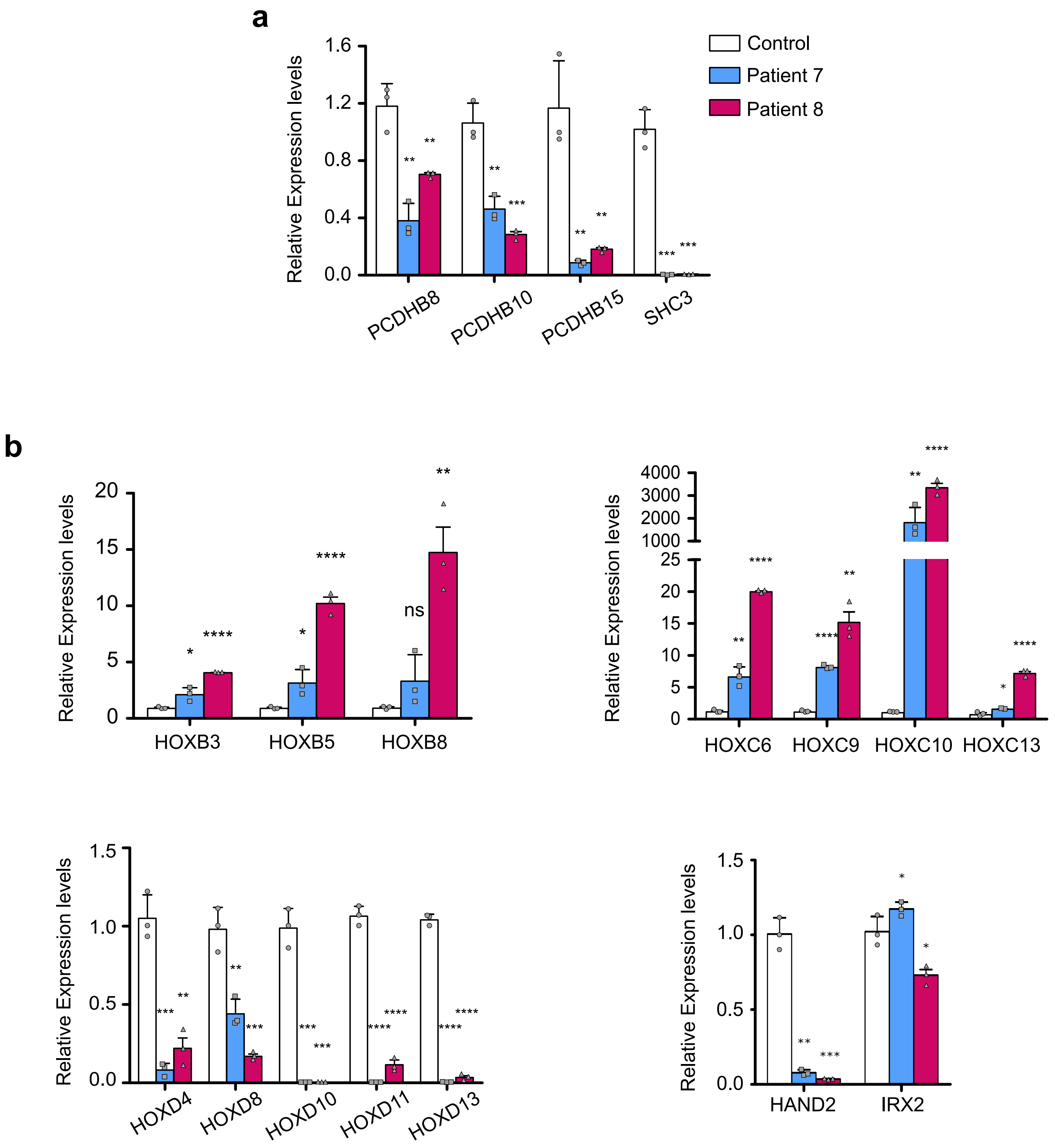

Supplementary Figure 4. Genes involved in development and differentiation are deregulated in *SMC1A*-deficient derived fibroblast. The mRNA expression levels of nervous system development genes **(a)** and embryonic development genes **(b)** were analyzed in a control (white) and two CdLS patient-derived fibroblast (P7, blue; P8, purple) with mutated-*SMC1A* by reverse transcription-qPCR. The graphs show the amount of transcript of each gene relative to that in the control. Means and SEMs were calculated from biological triplicates. Two-sided unpaired student's t-test (**** *p* <0.0001; *** *p* <0.001; ** *p* <0.01; * *p* <0.05).

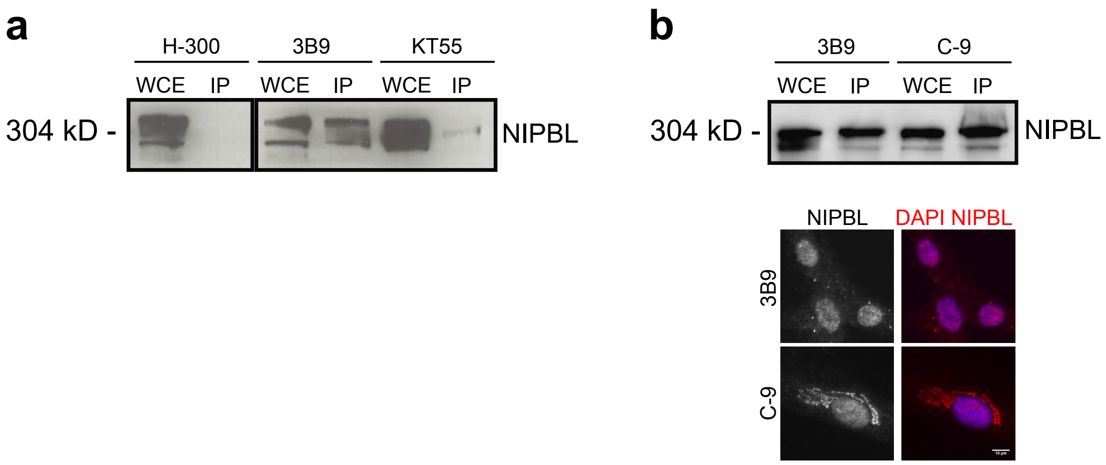

Supplementary Figure 5. Characterization of NIPBL antibodies. (a) Immunoprecipitation assays with three NIPBL commercial antibodies. Whole-cell extracts (WCEs) and the immunoprecipitation (IP) are shown. Representative blots from one biological triplicate are depicted. **(b, upper panel)** Immunoprecipitation assays with two commercial NIPBL antibodies, WCEs and the IP are shown. Representative blots from one biological triplicate are depicted. **(b, bottom panel)** Localization of NIPBL (grey channel) was monitored by immunofluorescence using two different NIPBL antibodies in control fibroblasts. Nuclei were stained using DAPI and merge signal (blue channel+ red channel) is shown. Scale bar, 10 μm. Abnormal NIPBL localization is observed using the C-9 antibody. Representative images from one biological triplicate are shown.

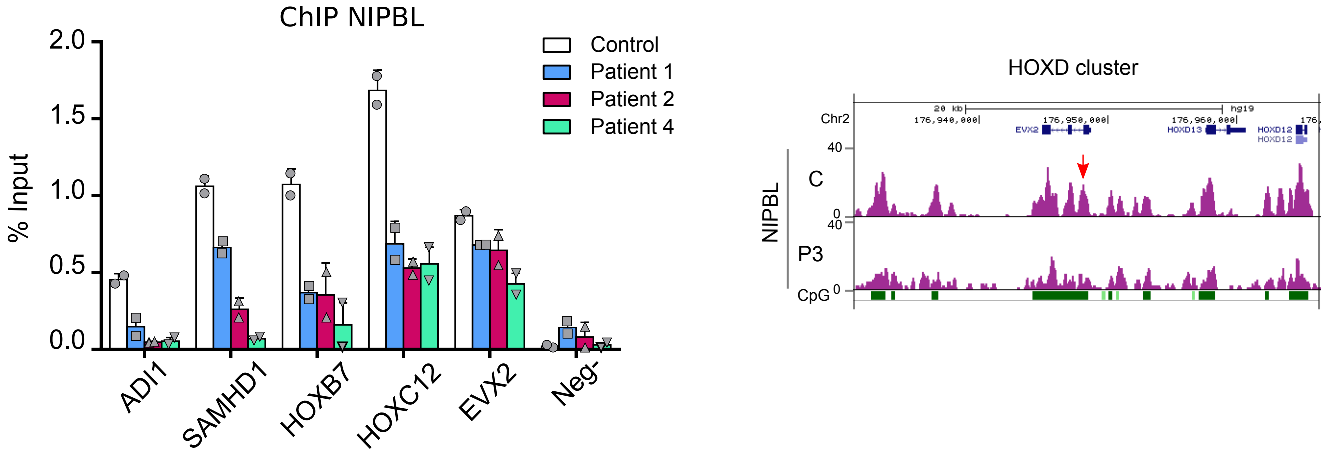

Supplementary Figure 6. Validation of the reduction of NIPBL peaks by ChIP-qPCR experiments. Cells from a control (white) and three CdLS-patients (P1, blue; P2, purple; P4, green) were used in ChIP-qPCR experiments to validate the NIPBL ChIP-Seq data using primers at the indicated genes. An intergenic region was used as a negative control (Neg -). Means and SEMs were calculated from biological duplicates (left). Snapshots of NIPBL peak distributions in control (C) and CdLS patient 3 cells around the HOXD cluster (right). The red arrows indicate the validated NIPBL peak around *EVX2* gene.

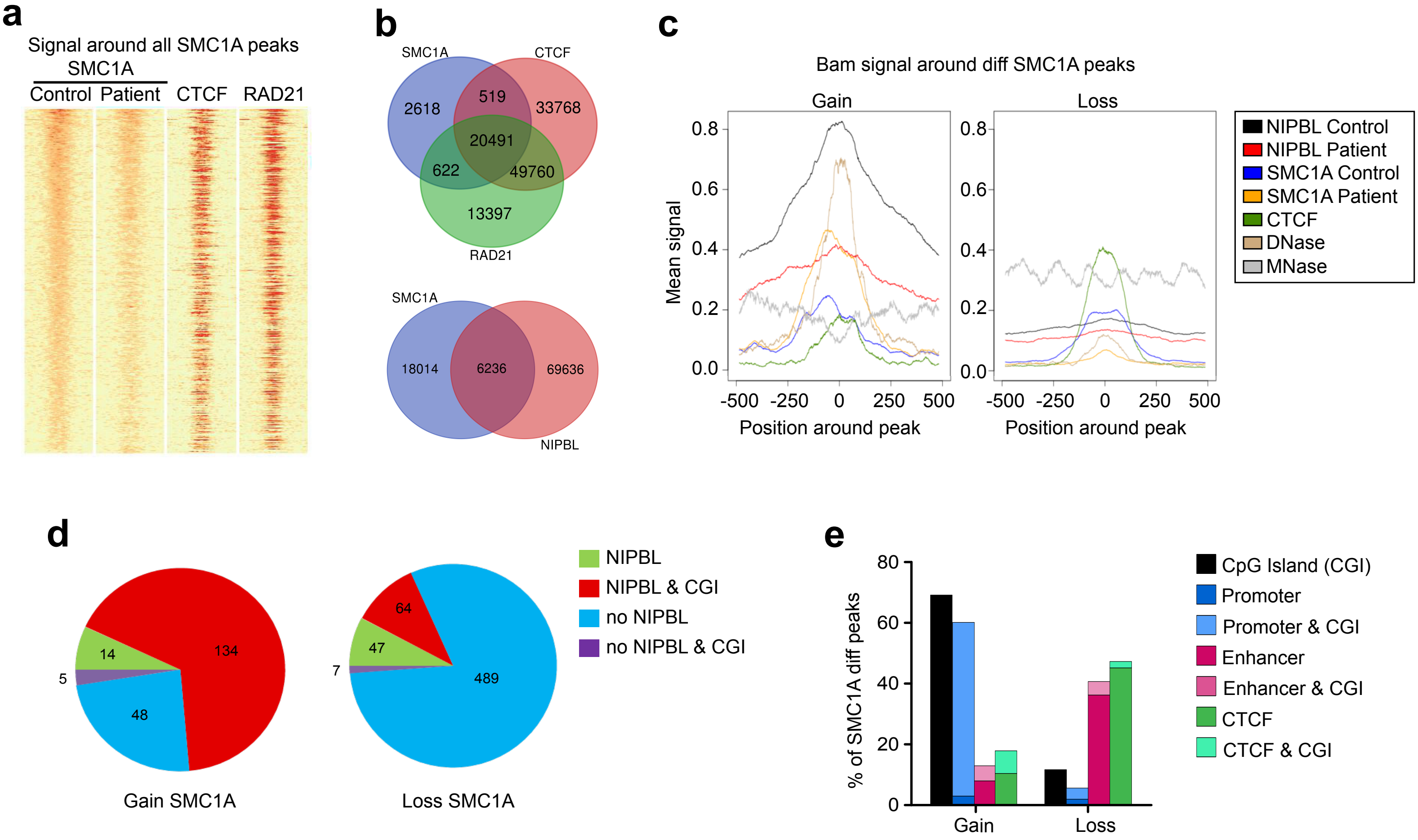

Supplementary Figure 7. Genome-wide distribution of SMC1A in Patient 4-derived cells. (a) Heatmap showing the signal around all SMC1A peaks in control and CdLS patient 4-derived cells and its co-localization with CTCF and RAD21 data from Encode. **(b)** Venn diagrams of SMC1A (dark blue) overlapping with CTCF (light red) and RAD21 (green) (upper) or SMC1A (dark blue) with NIPBL (light red) (bottom). **(c)** Bam graphs showing mean signal of the indicated proteins around gained (left) and lost (right) SMC1A differential peaks. NIPBL (Control, black; Patient, red), SMC1A (Control, blue; Patient, orange), CTCF (green) and MNase (grey). **(d)** Pie charts representing the overlap of gained (left) and lost (right) SMC1A peaks with NIPBL and CpG islands (CGI). NIPBL, green; NIPBL&CGI, red; no NIPBL, blue; no NIPBL&CGI, violet. **(e)** Plot showing the percentage of SMC1A gained and lost peaks that overlap with CpG islands (CGI, black), promoters (blue), enhancers (pink) and CTCF (green).

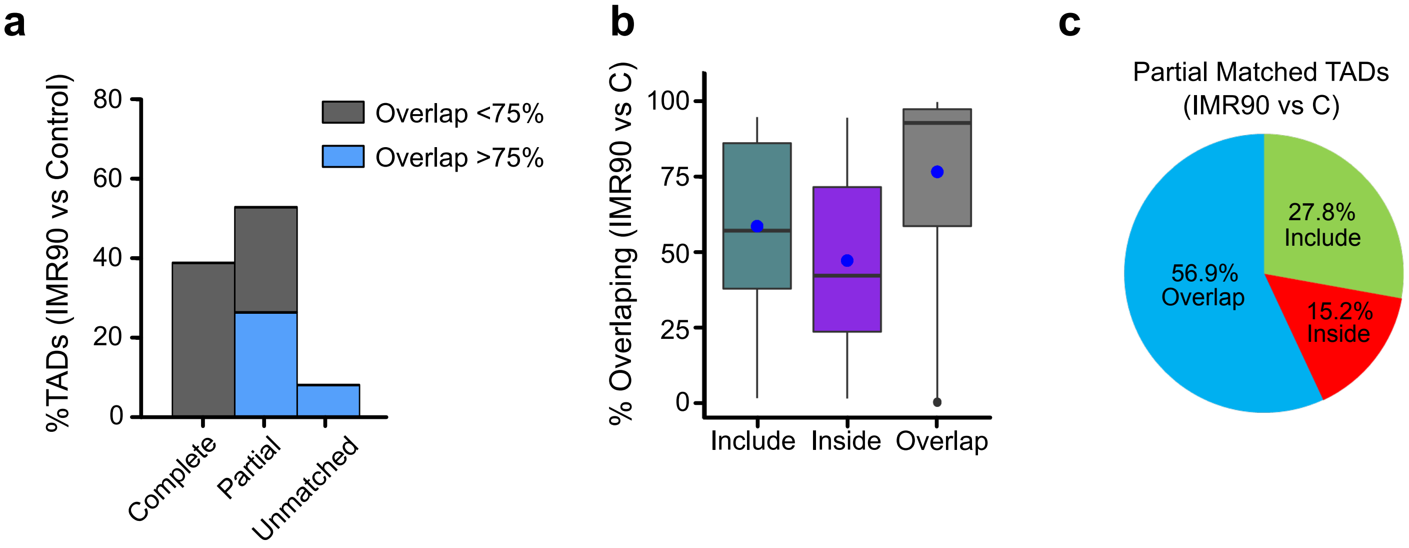

Supplementary Figure 8. Comparison of the intra-TAD prediction tool with Hi-C data. (a) Bar plots showing the percentage of experimental loops from IMR90 completely, partially matching or unmatching with the predicted intra-TAD loops from the control sample. Complete matching refers to a minimum of 95% reciprocal overlapping. Partial matching refers to full inclusion or partial overlap. The rest are unmatched TADs. TADs with an overlap of more than 75% (grey) are considered unchanged between the experimental and predicted loops. TADs with an overlap lower than 75% are considered different between the control and the patients (blue). **(b)** Boxplots showing the overlap percentage for partially matching TADs from (a) classified into include (dark green), inside (violet) and overlap (grey). Blue dots indicate the mean value in each case. Boxplots: center line, median; box limits, first and third quartiles; whiskers, 1.5x interquartile; points, outliers. **(c)** Pie plot representing the partial matching TADs from (a) classified into inside (red), include (green) and overlap (blue). 'Inside' refers to control predicted intra-TADs loops from the control residing inside an experimental loop, 'include' refers to control predicted intra-TADs loops including an experimental loop and 'overlap' refers to partial overlapping between both TADs.

Supplementary Table 1. Summary of the clinical and molecular features on the CdLS patients in this study

	Patient 1	Patient 2	Patient 3	Patient 4	Patient 5	Patient 6	Patient 7	Patient 8	Control 1	Control 2	Control 3	Control 4
**Gene Mutation**	NIPBL c.6647A>G p.Y2216C	NIPBL c.8387A>G p.Y2796C	NIPBL c.6647A>C p.Y2216S	NIPBL c.5689_5691delAAT p.N1897del	NIPBL c.6242G>C p.G2081A	NIPBL c.8387A>G p.Y2796C	SMC1A c.587 G>A p.R196H	SMC1A c.2096G>T p.R699L	–	–	–	–
**Age (years)**	2	10	5	16	11	14	10	5	3	11	30	30
**Clinical Score**	**16**	**5**	**15**	**13**	**14**	**11**	**13**	**5**				

Clinical Score ≥11 points, classic CdLS
9-10 points, non-classic CdLs
4-8 points, molecular testing
4 points, insufficient to indicate molecular testing CdLS

Supplementary Table 2. Gene ontology analysis of the differential expression genes in CdLS-derived cells. A pre-ranked Gene Set Enrichment Analysis (GSEA) was performed to separate upregulated biological processes in patients from those downregulated in patients. The log fold change values resulting from the DE analysis were used to rank the list of genes. GSEA software was run using 1000 gene permutations and a weighted gene enrichment statistic. Gene sets were limited to those containing between 15 and 500 genes (4116 gene sets). Gene sets with an FDR q-value < 0.25 were considered significantly enriched.

Downregulated genes

NAME	SIZE	ES	NOM p-val	FDR q-val
GO_AXIS_ELONGATION	30	-0,6684479	0	0,12885292
GO_REGULATION_OF_EPITHELIAL_TUBE_FORMATION	18	-0,7756181	0	0,1659859
GO_CHONDROCYTE_DEVELOPMENT	30	-0,6575674	0	0,16603169
GO_REGULATION_OF_ANIMAL_ORGAN_FORMATION	37	-0,5755641	0	0,18566899
GO_POSITIVE_REGULATION_OF_RHO_PROTEIN_SIGNAL_TRANSDUCTION	32	-0,6539717	0	0,19572045
GO_POSITIVE_REGULATION_OF_EPITHELIAL_CELL_DIFFERENTIATION	60	-0,5464879	0	0,2096403
GO_POSITIVE_REGULATION_OF_MESENCHYMAL_CELL_PROLIFERATION	25	-0,6623802	0	0,21004179
GO_NEGATIVE_REGULATION_OF_INTERLEUKIN_10_PRODUCTION	18	-0,6992185	0	0,23465733
GO_CELLULAR_RESPONSE_TO_VITAMIN	30	-0,6213291	0	0,23852745
GO_KIDNEY_MESENCHYME_DEVELOPMENT	19	-0,6903164	0,002702703	0,24239531
GO_ESTABLISHMENT_OF_PLANAR_POLARITY_OF_EMBRYONIC_EPITHELIUM	17	-0,7012875	0,002747253	0,19933875
GO_LYMPHANGIOGENESIS	15	-0,7596826	0,002857143	0,23817718
GO_SMOOTH_MUSCLE_TISSUE_DEVELOPMENT	22	-0,7045883	0,002906977	0,18820022
GO_REGULATION_OF_WATER_LOSS_VIA_SKIN	28	-0,6244028	0,003003003	0,21334948
GO_POSITIVE_REGULATION_OF_CARTILAGE_DEVELOPMENT	32	-0,5949158	0,003412969	0,22111532
GO_POSITIVE_REGULATION_OF_EPIDERMIS_DEVELOPMENT	38	-0,5666513	0,003745318	0,22062744
GO_ESTABLISHMENT_OF_PLANAR_POLARITY_INVOLVED_IN_NEURAL_TUBE_CLOSURE	15	-0,7450533	0,005181347	0,22725418
GO_COCHLEA_MORPHOGENESIS	24	-0,6434203	0,005830904	0,19489242
GO_BRANCHED_CHAIN_AMINO_ACID_METABOLIC_PROCESS	25	-0,6265594	0,006269592	0,20241025
GO_MALE_GENITALIA_DEVELOPMENT	23	-0,6472099	0,006309148	0,19277942
GO_BRANCH_ELONGATION_OF_AN_EPITHELIUM	19	-0,6843689	0,008426966	0,22365835
GO_METANEPHRIC_MESENCHYME_DEVELOPMENT	15	-0,7078603	0,012376238	0,21861541

Upregulated genes

NAME	SIZE	ES	NOM p-val	FDR q-val
GO_TRANSLATIONAL_INITIATION	186	0,6677141	0	0,00100415
GO_COTRANSLATIONAL_PROTEIN_TARGETING_TO_MEMBRANE	101	0,70649254	0	0,00149344
GO_ESTABLISHMENT_OF_PROTEIN_LOCALIZATION_TO_ENDOPLASMIC_RETICULUM	114	0,6835136	0	0,0039791
GO_NUCLEAR_TRANSCRIBED_MRNA_CATABOLIC_PROCESS_NONSENSE_MEDIATED_DECAY	119	0,6526558	0	0,02491821
GO_PROTEIN_LOCALIZATION_TO_ENDOPLASMIC_RETICULUM	142	0,63437	0	0,04248601
GO_PROTEIN_TARGETING_TO_MEMBRANE	200	0,6028843	0	0,09762804
GO_ANTIGEN_PROCESSING_AND_PRESENTATION_OF_EXOGENOUS_PEPTIDE_ANTIGEN_VIA_MHC_	79	0,65239376	0	0,10408586
GO_ANTIGEN_PROCESSING_AND_PRESENTATION_OF_PEPTIDE_ANTIGEN_VIA_MHC_CLASS_I	98	0,6297476	0	0,13729887
GO_ANTIGEN_PROCESSING_AND_PRESENTATION_OF_ENDOGENOUS_ANTIGEN	23	0,7702922	0	0,1576319
GO_NUCLEAR_TRANSCRIBED_MRNA_CATABOLIC_PROCESS	206	0,5899598	0	0,15863375
GO_MITOTIC_SPINDLE_ORGANIZATION	117	0,6093301	0	0,18598533
GO_CYTOPLASMIC_TRANSLATION	97	0,62129056	0	0,19238794
GO_RESPONSE_TO_INTERLEUKIN_12	50	0,6585492	0	0,20237195
GO_NEGATIVE_REGULATION_OF_NATURAL_KILLER_CELL_MEDIATED_IMMUNITY	17	0,7722237	0	0,20947562
GO_VIRAL_GENE_EXPRESSION	191	0,5818275	0	0,2116505
GO_ANAPHASE_PROMOTING_COMPLEX_DEPENDENT_CATABOLIC_PROCESS	82	0,6220927	0	0,2167282
GO_RNA_CATABOLIC_PROCESS	408	0,54527813	0	0,23730034
GO_T_CELL_RECEPTOR_SIGNALING_PATHWAY	197	0,5652017	0	0,24210757

Supplementary Table 3: List of primers used in this study

Name	Sequence	Use
PCDHB8 FW	CGAATGAGTTCCAGTTCCTGA	Expression
PCDHB8 RV	CTGAAACCAAAGCCATTCCT	Expression
PCDHB10 FW	CGGGACCAGTGAGTTCAAGT	Expression
PCDHB10 RV	TCCAAAGCTATTTCGGAAGG	Expression
PCDHB15 FW	CCCAAATATTGTAAGCCAGGA	Expression
PCDHB15 RV	AACAGACGGTCGGAAAGCTA	Expression
HOXB3 FW	CTTTTCTGCCTCGTCCTGTC	Expression
HOXB3 RV	ATTGGAGATGACCCCATCAA	Expression
HOXB5 FW	CGCTGGCCTCGTCTATTTC	Expression
HOXB5 RV	CAAGCCCTCTGCTTCGTC	Expression
HOXB8 FW	CAAGCTTGCAGTCTGCGTA	Expression
HOXB8 RV	GGCAATTTCTACGGCTACGA	Expression
HOXC6 FW	CAGACCCTGGAACTGGAGAA	Expression
HOXC6 RV	ATCTGTCGCTCGGTCAGG	Expression
HOXC9 FW	ACCTCCTAGCGTCCAGGTTT	Expression
HOXC9 FW	CGAAGCTACAGGACGGAAAA	Expression
HOXC10 FW	AACATCTGGAATCGCCTCAG	Expression
HOXC10 RV	GCTCTGCTCCGTCTTGATTT	Expression
HOXC13 FW	CTTCGGGGGCAGCTACTAC	Expression
HOXC13 RV	GACGGCTCCGGGTATTTATC	Expression
HOXD4 FW	CAGGGCGCCGACTACTAC	Expression
HOXD4 RV	AAAGGCTGCTCACCGAAGT	Expression
HOXD8 FW	CCCTCACCGAGAGACAGGTA	Expression
HOXD8 RV	CGGGAAATTTGTCCTTGTTG	Expression
HOXD10 FW	GTTCCCGTCCCTGGATATTT	Expression
HOXD10 RV	GTTCCCGTCCCTGGATATTT	Expression
HOXD11 FW	GACTTCGCTAGCAAGCCTTC	Expression
HOXD11 RV	GGAGCCAGGTTGGAAGAGTA	Expression
HOXD13 FW	CTCCCCCAGCCAAAGAGT	Expression
HOXD13 RV	GCCGAAGTGGTAGCCGTAG	Expression
HAND2 FW	ATACTCGGGGCTGTAGGACA	Expression
HAND2 RV	GCCATGAGGAGAACCCCTAC	Expression
IRX2 FW	GCCCAGGTCATCTTGTTCTC	Expression
IRX2 RV	TGCTAGCCATCATCACCAAG	Expression
ANKRD26 FW	AATCTCTCCCTCCGGGTTAC	SMC1A ChIP
ANKRD26 RV	CATGGAGCACACTTGACCAC	SMC1A ChIP
ACTN4 FW	CGGGCTGAAGCAGCTGAA	SMC1A ChIP
ACTN4 RV	GCCGTACTGGTACGACTGGT	SMC1A ChIP
CBX5 FW	CGTCTGCCCTGTTCCTGTAT	SMC1A ChIP
CBX5 RV	GCGGGAGTTTGTCCTAGCTT	SMC1A ChIP
PHF12 FW	AGCGCACTGAGACAAAAGG	SMC1A ChIP
PHF12 RV	TTTGCACACAAACACCAATG	SMC1A ChIP
TUBD1 FW	TATGGTACGCATGCTCACTG	SMC1A ChIP
TUBD1 RV	GTGGACATGCGCAGAACTAT	SMC1A ChIP
LOC100507642 FW	ACGCCATAGTCACCTCATCA	SMC1A ChIP
LOC100507642 RV	GAAGACAATGGGGGAGGAC	SMC1A ChIP
FAM98A FW	GGAACTTCCAAATCCACAGC	SMC1A ChIP
FAM98A RV	CGGGCCCTGTAATTCTTTAG	SMC1A ChIP
CCDC12 FW	CGGCAGCTGTCTTTCCTTAT	SMC1A ChIP
CCDC12 RV	GCAAGGTGAGAAGTGTGGAG	SMC1A ChIP
ADI1 FW	ACACCCCAGGCAACAGAAAC	SMC1A & NIPBL ChIP
ADI1 RV	AGCGAAGTTTACGGGGTTG	SMC1A & NIPBL ChIP
N6AMT1 FW	GCTCGTACACGTCGCTGAAG	SMC1A ChIP
N6AMT1 RV	GAACGCAGCGAAGGACTATG	SMC1A ChIP
Neg- FW	AAAGTGGCACCGTCGTAAAG	SMC1A & NIPBL ChIP
Neg- RV	CCTATCCTCACCCCCATTTT	SMC1A & NIPBL ChIP
HOXB4 FW	CACAGGGCTAGAGGGAGAGTT	SMC1A ChIP
HOXB4 RV	CCGCCTTAGACCAAGTTCAC	SMC1A ChIP
HOXB5 ChIP FW	GAGAATCGTTAGGGCCGATT	SMC1A ChIP
HOXB5 ChIP RV	GCCAAAGCCAACCTTCTCTC	SMC1A ChIP
HOXB8 ChIP FW	CTCCAGGGAGTTTCACATGG	SMC1A ChIP
HOXB8 ChIP RV	GCTGCTCTCCCAGAAAACTG	SMC1A ChIP
HOXC5 FW	CCCCAATTCCAAGAACCTTT	SMC1A ChIP
HOXC5 RV	TCTCGATATGTTGGCAAAGC	SMC1A ChIP
HOXC8 FW	GAGGTTCCTTCCTCCCACTT	SMC1A ChIP
HOXC8 RV	CATTTGGTCCAATGCAGTCC	SMC1A ChIP
EVX2 FW	CGGGCACACTCAGAGAGG	SMC1A ChIP
EVX2 RV	TTAGTAAGACGGCCGTGAGC	SMC1A ChIP
HOXD4 ChIP FW	TTCCCGCGCACTCTACCT	SMC1A ChIP
HOXD4 ChIP RV	CAGTCTGGGGAAAGGAACC	SMC1A ChIP
PCDHB16 FW	GCGCTGCAGGTTAAGATGAC	SMC1A ChIP
PCDHB16 RV	CGCTCTGGCTCAGCTATTCT	SMC1A ChIP
PCDHB10 ChIP FW	CACACACCCAAATGGGTACA	SMC1A ChIP
PCDHB10 ChIP RV	CTCAGAGCAATTCCCAAACC	SMC1A ChIP
PCDHB13 FW	GTGCATACAGGCAGAGTTGG	SMC1A ChIP
PCDHB13 RV	GGTTCTGGTTTGTGCCTTCT	SMC1A ChIP
PCDHB14 FW	GGTGGCGCTACAGGATAAGA	SMC1A ChIP
PCDHB14 RV	GTGGGTTGGATGCTTTCTGT	SMC1A ChIP
TAD1 FW	GCATTGCTTTGCTAGCTCCT	3C
TAD1 RV	GCCTCCCAAAGTGCTGGTAT	3C
TAD2 FW	AGATGACAGGCTTTGCTGCT	3C
TAD2 RV	GGCTGGCAGAGACAGAGAGA	3C
TAD3 FW	CCTACCCACTCACCTCCACA	3C
TAD3 RV	AAGAGCGAAACTCCGTCTCA	3C
TAD4 FW	AATAGGGCACTGAAGGATGG	3C
TAD4 RV	CTTGGCCTTCCGAAGTTCT	3C
TAD5 FW	TGTGCAAACTCTGACCTGCT	3C
TAD5 RV	GAACTAGGCCACCCAGCAG	3C
TAD6 FW	AGAAACCAGGGACACACAGC	3C
TAD6 RV	AGGTGGGATGAGTTGGTGAG	3C
SAMHD1 FW	ACTGACAGTTGAGCCCTTCG	NIPBL ChIP
SAMHD1 RV	GGGATTGATTTGAGGACGAC	NIPBL ChIP
HOXB7 FW	CAGGGGTAGATCCGGAAGTT	NIPBL ChIP
HOXB7 RV	GCCCTTTGAGCAGAACCTCT	NIPBL ChIP
HOXC12 FW	CGGCTTCAAGTACGACTACG	NIPBL ChIP
HOXC12 RV	GAGTCGGATTCCAGCGACT	NIPBL ChIP
EVX2 ChIP FW	TGCTAGCTCGGTGTAGCTTG	NIPBL ChIP
EVX2 ChIP RV	CAGTCCCATCCTCTTTGCTC	NIPBL ChIP
