# Supplementary material for: Disruption of NIPBL/Scc2 in Cornelia de Lange Syndrome provokes cohesin genome-wide redistribution with an impact in the transcriptome
Source: Nat Commun. 2021 Jul 27;12:4551. doi: 10.1038/s41467-021-24808-z (PMC8316422; doi:10.1038/s41467-021-24808-z)
Supplement: Supplementary file 3 — Description of Additional Supplementary Files [file 41467_2021_24808_MOESM3_ESM.pdf]

Description of Additional Supplementary Files

File Name: **Supplementary Data 1**.
Description: **Gene expression raw data in CdLS-derived cells.**

File Name: **Supplementary Data 2**.
Description: **Differential expression genes in CdLS-derived cells.** The medians of pixels intensity were background corrected and normalized between arrays. The selection of differential expressed genes (DEG) was based in a linear model adjusted by age stages. Two age stages were considered, individuals under and over 15 years old. Computation of *p*-values was performed by moderated t-statistics using empirical Bayes shrinkage and adjusted to control the False Discovery Rate using the Benjamini and Hochberg method. Genes were considered DEG when $FDR<0.25$ and $|logFC| >0.5$.

File Name: **Supplementary Data 3**.
Description: **Peak calling of NIPBL spike-in ChIP-seq.**

File Name: **Supplementary Data 4.**
Description: **Differential methylated positions in CdLS-derived cells.** Quantile and functional normalization were performed using the minfi package in R. Probes located on X/Y chromosomes and SNPs were excluded to avoid biological and technical biases. Signal intensities against background noise were compared and excluded those CpGs with values of $p > 0.01$ in more than one sample. A linear model was derived using minfi for the differential DNA methylation analysis and the resulting probabilities were corrected for multiple testing (FDR). CpGs were considered differential methylated when $FDR < 0.05$.

File Name: **Supplementary Data 5.**
Description: **Peak calling of SMC1A ChIP-seq.**

File Name: **Supplementary Data 6.**
Description: **Differential SMC1A peaks.**

File Name: **Supplementary Data 7**.
Description: **Peak calling of SMC1A ChIP-seq, patient 4.**

File Name: **Supplementary Data 8**.
Description: **Differential SMC1A peaks, patient 4.**

File Name: **Supplementary Data 9.**
Description: **Gene ontology of the differential SMC1A peaks.** The gene ontology biology processes were obtained using DAVID functional annotation bioinformatics. Multiple testing correction using Bonferroni, Benjamini and FDR are shown. Gene sets with an FDR q-value <0.05 were considered significantly enriched (marked in blue).

File Name: **Supplementary Data 10.**
Description: **Comparison of the intra-TAD prediction tool with Hi-C data from Rao *et al.*[78] (a)** Summary of the number of loops with an identified CTCF motif in both anchors for the IMR90 cell lines based on the CTCF motif search used in the intra-TAD prediction tool (this study) and the original Hi-C data[78]. **(b)** Summary of the loops from the experimental Hi-C data (IMR90 cell line) compared with the intra-TAD prediction tool (*in-silico*) using our control fibroblast ChIP-seq data. We quantified the *in-silico* and experimental TADs that reciprocally overlapped in a 95% minimum and categorized them as a complete match. We considered a partial match when (i) an *in-silico* TAD fully included an experimental TAD (*include*) or vice versa (*inside*), and (ii) an *in-silico* and experimental TADs partially overlapped. The rest of *in-silico* or experimental TADs were labelled as unmatched TADs.

File Name: **Supplementary Data 11.**
Description: **List of TADs predicted from the control and patients-derived fibroblast ChIP-seq data using the intra-TAD prediction tool. (a)** Summary of number of TAD matches among experiments and TADs predicted in Control and Patients. **(b-e)** Complete list of predicted intra-TADs for control fibroblast **(b)**, both patients analysed together **(c)** or individually Patient 3 **(d)** and Patient 4 **(e)**.
